# Esophageal dysmotility in patients with eosinophilic esophagitis: pathogenesis, assessment tools, manometric characteristics, and clinical implications

**DOI:** 10.1007/s10388-022-00964-z

**Published:** 2022-10-11

**Authors:** Pierfrancesco Visaggi, Matteo Ghisa, Elisa Marabotto, Arianna Venturini, Delio Stefani Donati, Massimo Bellini, Vincenzo Savarino, Nicola de Bortoli, Edoardo Savarino

**Affiliations:** 1 Gastroenterology Unit, Department of Translational Research and New Technologies in Medicine and Surgery, University of Pisa, Pisa, Italy; 2 Gastroenterology Unit, Department of Surgery, Oncology and Gastroenterology, University of Padua, Via Giustiniani 2, 35128 Padua, Italy; 3 Gastroenterology Unit, Department of Internal Medicine, University of Genoa, Genoa, Italy

**Keywords:** Eosinophilic esophagitis, Esophageal motility, Diagnosis

## Abstract

Eosinophilic esophagitis (EoE) represents a growing cause of chronic esophageal morbidity whose incidence and prevalence are increasing rapidly. The disease is characterized by eosinophilic infiltrates of the esophagus and organ dysfunction. Typical symptoms include dysphagia, chest pain, and bolus impaction, which are associated to mechanical obstructions in most patients. However, up to one in three EoE patients has no visible obstruction, suggesting that a motor disorder of the esophagus may underlie symptoms. Although potentially relevant for treatment refractoriness and symptomatic burden, esophageal dysmotility is often neglected when assessing EoE patients. The first systematic review investigating esophageal motility patterns in patients with EoE was published only recently. Accordingly, we reviewed the pathogenesis, assessment tools, manometric characteristics, and clinical implications of dysmotility in patients with EoE to highlight its clinical relevance. In summary, eosinophils can influence the amplitude of esophageal contractions via different mechanisms. The prevalence of dysmotility may increase with disease duration, possibly representing a late feature of EoE. Patients with EoE may display a wide range of motility disorders and possible disease-specific manometric pressurization patterns may be useful for raising a clinical suspicion. Intermittent dysmotility events have been found to correlate with symptoms on prolonged esophageal manometry, although high-resolution manometry studies have reported inconsistent results, possibly due to the suboptimal sensitivity of current manometry protocols. Motor abnormalities may recover following EoE treatment in a subset of patients, but invasive management of the motor disorder is required in some instances. In conclusion, esophageal motor abnormalities may have a role in eliciting symptoms, raising clinical suspicion, and influencing treatment outcome in EoE. The assessment of esophageal motility appears valuable in the EoE setting.

## Introduction

Eosinophilic esophagitis (EoE) is an emerging disease whose incidence and prevalence have risen steadily over the past three decades [1, 2]. Nevertheless, it is estimated that EoE is diagnosed with a mean delay of up to 10 years [3, 4]. The disease is characterized by a chronic eosinophil-predominant inflammation restricted to the esophagus triggered by the exposure to food or inhalant antigens, which causes symptoms of esophageal dysfunction [5]. The diagnosis is made based on the presence of at least 15 eosinophils/HPF (high-power field) in at least one esophageal biopsy in combination with a suggestive clinical history, following the exclusion of secondary causes of eosinophilia [6, 7]. Common presenting symptoms include dysphagia, chest pain, and episodes of food bolus impaction which, in most patients, are related inflammatory or fibrotic obstructions of the esophagus. In particular, EoE patients may have esophageal edema, transient or fixed rings, white plaques or exudates, linear furrows, or overt fibrotic stenoses on endoscopy [8]. Although mechanical obstructions represent a major cause for the pathogenesis of symptoms, one in three EoE patients has a macroscopically normal upper endoscopy but experiences obstructive symptoms nevertheless, indicating that the origin of symptoms may be functional in such patients [9]. In this regard, eosinophilic and mast cells products have been shown to affect visceral motility and function in EoE via several mechanisms [10]. Additionally, there is modest correlation among histology, endoscopy, and clinical disease activity in EoE [5, 11] and esophageal dysmotility may be a contributing factor to this discrepancy.

A recent systematic review on ongoing clinical trials on the treatment of EoE found that esophageal motility is not routinely assessed in the setting of EoE clinical trials [12]. Although EoE is a relatively recent disease, the knowledge on its pathophysiology is growing at a fast rate, and evidence on the pathogenesis, techniques for assessment, manometric characteristics, and clinical implications of dysmotility in patients with EoE is becoming available. Accordingly, we performed a review of the literature to summarize current knowledge on esophageal dysmotility in EoE.

### Literature research and eligibility criteria

According to the aim of this narrative review, we reviewed randomized controlled trials (RCT), original articles, and conference papers reporting on esophageal motility in patients with EoE. We conducted a literature review using the electronic databases PubMed/MEDLINE and the Cochrane Library. Search terms used included eosinophilic esophagitis, esophageal motility, dysmotility, motor findings, pathogenesis, diagnosis, treatment, fibrosis, dysphagia, food impaction, stricture, esophageal pressurization, esophageal manometry, high-resolution manometry, conventional manometry, standard manometry, and manometry. The term eosinophilic esophagitis was used as MeSH term. Two authors (PV, MG) independently reviewed all manuscripts published from inception to July 2022. Papers defining EoE as the presence of at least 15 eosinophils per high-power field in at least one esophageal biopsy in combination with symptoms of esophageal dysfunction and studies assessing esophageal motility on esophageal manometry were eligible for inclusion. All papers were included based on a consensus decision of scientific merit by the reviewing authors.

### Pathogenesis of motor abnormalities in EoE

Eosinophils are pleiotropic leukocytes involved in initiation and propagation of inflammatory responses, whose activation and degranulation releases cytotoxic granules containing cationic proteins, namely major basic protein (MBP), eosinophil cationic protein, eosinophil peroxidase, and eosinophil-derived neurotoxin, which induce tissue damage and dysfunction [13]. In patients with EoE, the uncontrolled transmural inflammation of the esophagus drives a progressive remodeling of the esophageal wall with fibrosis of the lamina propria, basal cells hyperplasia, epithelial mesenchymal transition, sub-epithelial angiogenesis, and smooth muscle hypertrophy [14, 15]. These structural modifications ultimately result in impaired esophageal function with reduced wall compliance and altered contractility caused by transmural rigidity and stiffening [16]. Of note, distensibility and endoscopic features can improve following successful EoE treatment [17] but worsen with disease progression and increasing age [18].

Along with the biomechanical implications of remodeling, eosinophilic products have a neurotoxic activity, which can modify the amplitude of esophageal muscle contractions by influencing the local release of neurotransmitters to Auerbach’s myenteric and Meissner’s submucosal nervous plexuses [19, 20]. For example, the MBP activates muscarinic M2 acetylcholine receptors and stimulates the contraction of smooth muscles in the distal two thirds of the esophagus, while eosinophilic interleukins inhibit the release of acetylcholine, reducing the contractility of smooth muscle cells [21]. Additionally, eosinophil degranulation has been shown to induce axonal necrosis [22], which impairs the effective delivery of neurotransmitters to the esophagus. In support of the causative role of eosinophils in motor disturbances, dense eosinophilic infiltrates have been found in the esophageal muscular layers of patients with esophageal hypercontractility [23] or gastric dysmotility [24]. Further supporting this concept is the established association of esophageal eosinophilia and achalasia [25]. Although food stasis might contribute to esophageal eosinophilia in achalasia patients, eosinophilia may not resolve or may increase postoperatively [26, 27].

Unlike eosinophils, mast cells are present in the esophageal mucosa and submucosa of healthy individuals; however, upregulation of mast cells-specific genes and an increased number of IgE-bounded mast cells are found in esophageal biopsies of EoE patients [28]. Mast cells can induce muscle cells to differentiate into a more contractile phenotype, and release myoactive and neuroactive mediators that activate smooth muscle contraction pathways. Accordingly, recent studies suggested an eosinophil-independent role of mast cells in causing dysmotility in EoE [10]. Finally, although genetic susceptibility has a role in the pathogenesis of EoE and there is evidence of disease heritability [28], no studies have investigated the contribution of genetic variants to esophageal dysmotility in EoE to date.

### How to assess esophageal motility in EoE

Patients with an esophageal motor disorder usually complain of symptoms of esophageal dysfunction. As for other alarm symptoms related to the upper gastrointestinal tract, esophagogastroduodenoscopy (EGDS) should be the first step of the diagnostic algorithm to rule out malignancies [7]. Endoscopy does not allow to adequately assess esophageal motor function, although gross abnormalities of the esophageal motility as the absence of peristalsis or the presence of tertiary contractions can be identified through a careful observation of the esophageal body, even by non-expert endoscopists [29]. Consistently, an esophageal dilatation with tortuosity and retained saliva or food can suggest a diagnosis of achalasia in up to 30–50% of patients [30]. Recently, artificially intelligence (AI) tools have been applied to assist in the diagnosis of esophageal diseases [31]. However, a recent meta-analysis found that there are no studies investigating the use of AI for the diagnosis of dysmotility in EoE patients [32].

When the EGDS has ruled out malignancies or an alternative diagnosis that can explain symptoms, the physician has two aces to play: high-resolution manometry (HRM) and the barium esophagogram (BE). BE has been generally seen as a complementary test in the assessment of esophageal dysfunction. Data regarding the diagnostic value of BE in comparison with HRM are discordant. A study comparing BE with HRM for the diagnosis of achalasia reported a sensitivity, specificity, and accuracy of 78.3%, 88.0%, and 83.0%, respectively [33]. Other data showed that BE could accurately rule out achalasia, although it was significantly less able to diagnose other motor disorders [30].

The timed BE technique (TBE), which consists in repeating radiography at set time intervals after a barium swallow to assess esophageal emptying, is generally preferred both for the diagnosis of achalasia and the assessment of the response to treatments [30]. In a study comparing TBE with HRM, TBE showed sensitivity of 85% and specificity of 86% in differentiating untreated achalasia from EGJ-OO [34].

HRM took the field by the 90s and it is currently considered the gold standard for the assessment of esophageal body peristalsis and LES function. HRM has several advantages over conventional manometry (CM), including the ease of positioning the catheter, the reduced risk of displacement during the examination, and the increased number of pressure sensors. Additionally, in CM non-specific motility abnormalities are frequently reported without the possibility of reaching a definitive diagnosis [35]. The only prospective randomized trial comparing HRM and CM found a higher sensitivity of HRM for the diagnosis of achalasia, compared to CM (93% vs. 78%), with identical specificity (100%) [36].

In HRM, the data acquired from the manometry probe are represented as esophageal pressure topography plots or “Clouse Plots,” which use a color code to describe the peristaltic amplitude in a space–time continuum providing a seamless representation of the pressure activity through the swallow, making its interpretation easier.

One of the greatest advances in the evaluation of esophageal motility by HRM was the introduction of a standardized protocol, specific parameters for the interpretation, and clear diagnostic criteria for motor disorders. These achievements were made possible with the introduction of the CC, now at its fourth iteration (CCv4.0) [37]. A standard HRM protocol requires at least ten 5 mL water swallows, obtained in both supine and sitting position. Provocative tests (i.e., multiple rapid swallows—MRS and rapid drinking challenge—RDC) were included to investigate the peristaltic reserve and outflow obstruction [38, 39]. According to CCv4.0, the esophagogastric junction (EGJ) morphology and LES relaxation should be assessed first. In case of a non-relaxing LES (elevated median integrated relaxation pressure—IRP) combined with the absence of normal peristalsis, a diagnosis of achalasia can be made. Instead, EGJ outflow obstruction (EGJ-OO) is characterized by a non-relaxing LES with preserved esophageal body peristalsis. EGJ-OO should be considered clinically relevant and candidate for treatment only in case of elevated IRP in both supine and sitting position, together with dysphagia and/or non-cardiac chest pain, and when there is at least one adjunctive investigation indicating obstruction (e.g., TBE or EndoFLIP—Endolumenal Functional Lumen Imaging Probe) [39]. After the assessment of the EGJ, the esophageal body function should be evaluated. In patients with normal LES relaxation and 100% failed peristalsis after wet swallows, a diagnosis of absent peristalsis can be made. Instead, distal esophageal spasm (DES) is diagnosed when ≥ 20% of swallows are followed by premature contractions (distal latency—DL < 4.5 s). The presence of more than 20% of hypercontractile contractions (i.e., DCI >  = 8000 mmHg-s-cm) allows to reach the diagnosis of hypercontractile esophagus (HE). Of note, JE is no more a synonym of HE, but it represents a subtype of HE with multipeaked contractions [37]. As regards ineffective esophageal motility (IEM), a conclusive diagnosis requires more than 70% ineffective swallows (distal contractile integral—DCI < 450 mmHg-s-cm) or at least 50% of failed peristalsis (DCI < 100 mmHg-s-cm).

The latest advance in the assessment of esophageal motility is the esophageal impedance planimetry, a device commercially available as EndoFLIP. With this technique, the cross-sectional area of the esophagus is simultaneously measured at multiple levels using a saline-filled cylindrical bag containing an array of impedance electrodes. Available data from recent studies suggest that impedance planimetry may be a useful complementary diagnostic tool for the diagnosis of manometric disorders, especially in those with disorders of the EGJ outlet [39, 40].

### Characteristics of esophageal motility in EoE

Only recently, a systematic review of the literature investigating esophageal motility patterns in patients with EoE found that studies reporting on esophageal motility in EoE used heterogeneous manometry protocols and guidelines for the interpretation of tracings [41].

In 1978, Landres et al. [42] reported a case of a patient with vigorous achalasia who had conspicuous eosinophilic infiltration of the esophagus. At that time, EoE was not regarded as a distinct disease, and the authors concluded that such eosinophilic inflammation could represent a variant of the eosinophilic gastroenteritis syndrome and could predispose to esophageal motor disorders. In 1993, Attwood et al. [43] described 12 patients with esophageal eosinophilia, dysphagia, normal pH-metry (in 11/12 cases), and normal EGDS, 83% of which were found to have a non-specific motor disorder (NSMD) on CM. The authors concluded that the presence of high concentrations of eosinophils in esophageal biopsies in conjunction with dysphagia, normal endoscopy, and normal 24-h esophageal pH monitoring represented a distinctive clinicopathologic syndrome which had not been previously described. In 1996, Hempel et al. [44] provided the first report of a patient with non-cardiac chest pain, esophageal eosinophilic infiltrates, and tertiary contractions on CM whose dysmotility and symptoms recovered following corticosteroid therapy. Similarly, in 2006, Lucendo [45] reported a case of eosinophilic esophagitis with absent peristalsis on CM whose peristalsis recovered with 80% of normal-amplitude peristaltic waves following topical corticosteroid treatment. The following year, Lucendo et al. [46] corroborated these findings in a cohort of 12 EoE patients. Of these, 10 presented with NSMD or hypercontractility or primary simultaneous waves, and 3 had normal peristalsis at baseline CM. Following treatment with topical steroids, manometry, symptoms, and histology improved in 7/9 of patients with a previous diagnosis of dysmotility. Moawad et al. [47] retrospectively investigated CM findings in 75 EoE patients undergoing CM. In this cohort, 37% of patients had IEM or NE, whereas 63% had normal motility.

As regard children, Cheung et al. [48] retrospectively investigated esophageal motility in a cohort of 11 pediatric patients with EoE and found that 100% had normal peristalsis on CM off therapy. In contrast, another study on 17 EoE children demonstrated NSMD in 41% and normal motility in 59% of patients. However, when undergoing 24-h prolonged esophageal manometry all patients showed some evidence of dysmotility. Additionally, all episodes of dysphagia reported by patients during the prolonged manometric monitoring were associated to a simultaneous episode of dysmotility.

The first prospective study on esophageal motility in patients with EoE dates to 2009, when Bassett and colleagues [49] assessed the results of CM in EoE patients at a military treatment facility. The authors found that 23% of patients had a NSMD or high-amplitude peristaltic waves, whereas 77% had normal peristalsis. Similar results were prospectively achieved by Monnerat et al. [50]. The authors evaluated 20 EoE patients, of which 25% had IEM or LES dysfunction on CM, while 75% had normal peristalsis.

**Fig. 1:**
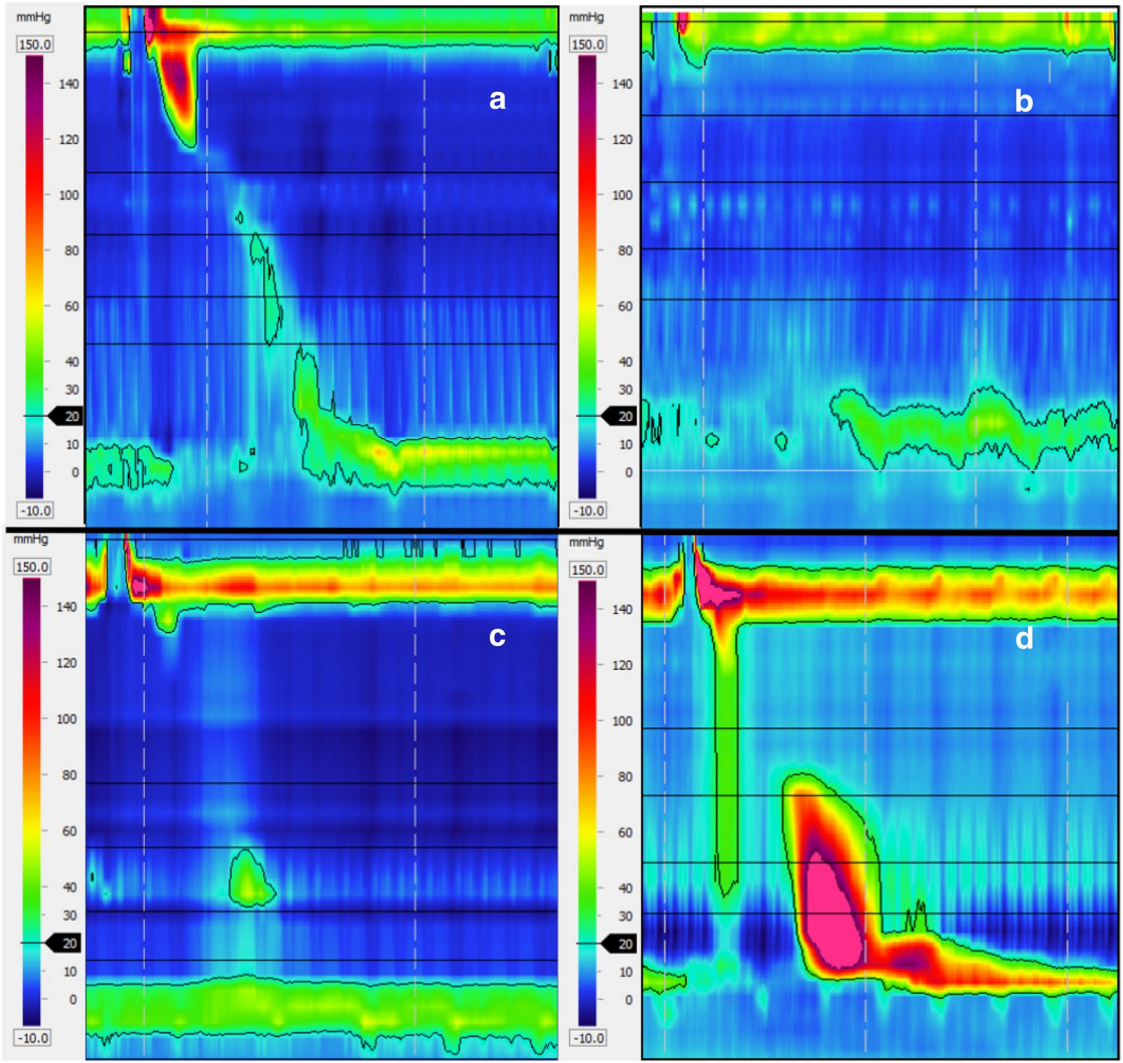
High-resolution manometry examples of motor patters commonly observed in patients with EoE: **a** ineffective esophageal motility; **b** absent peristalsis, with normal EGJ relaxation pressure; **c** absent peristalsis, with increased EGJ relaxation pressure (achalasia); **d** early esophageal pressurization

In recent years, several studies investigating esophageal motility in EoE patients by means of HRM have been published (Fig. 1). In 2011, Martin et al. [51] prospectively identified a series of esophageal motor abnormalities in 21 proton pump inhibitors (PPI)-nonresponsive patients with EoE undergoing HRM. The authors assessed HRM tracings according to CC v1.0 criteria [52]. Twenty-four percent of patients showed normal peristalsis, while 48% had pan-esophageal pressurization (PEP) (i.e., pressurization > 30 mmHg spanning from the upper esophageal sphincter to the LES), and 28% had peristaltic dysfunction. Interestingly, patients with PEP did not have visible endoscopic obstructions, suggesting that PEP represented an impaired bolus transit per se. Additionally, PEPs were found to statistically correlate with previous episodes of bolus impaction. In the same year, Roman et al. published a retrospective study on HRM in EoE [53]. Among 48 patients, 18 (37.5%) had evidence of dysmotility, while 30 (62.5%) had normal peristalsis. Of note, 36% of patients had PEP within two seconds of the esophageal contraction (i.e., early PEP) or compartmentalized distal pressurization (CDP) during single wet swallows. In another study, among a cohort of 20 EoE patients, 40% showed early PEP or CDP during single wet swallows. Following treatment with topical steroids, abnormal pressurizations disappeared in 86% of cases [54]. A comparable case was reported by Savarino et al. [55]. The authors described a patient with EoE and achalasia who demonstrated normal motility on HRM following treatment with systemic steroids. In contrast, topical and systemic steroids were not effective at managing a case of EoE with jackhammer esophagus, in which a per-oral endoscopic myotomy was necessary to achieve the control of esophageal symptoms [56].

In 2014, Clayton et al. [57] retrospectively found that an elevated IBP could distinguish the fibrostenotic from the inflammatory phenotype of EoE, having patients with fibrostenosis significantly higher values of IBP, which is consistent with a reduced compliance of the esophageal wall in these patients. Among 10 patients, the authors found that 33% had evidence of abnormal esophageal motility, while 67% had normal peristalsis at HRM. In contrast, von Arnim et al. [58] prospectively found that intrabolus pressure (IBP) was significantly higher in EoE patients compared to controls but could not discriminate EoE phenotypes in a cohort of 24 patients.

More recently, it has been reported that achalasia and obstructive motility disorders can be found in approximately 15% of patients with EoE [27]. These patients may not always respond to medical treatment and may require invasive management including laparoscopic Heller’s myotomy and/or pneumatic dilation for the resolution of dysphagia. Similarly, Savarino et al. [59] found that, in a cohort of 35 EoE patients, 17% had achalasia or obstructive motility disorders, 26% showed hypomotility disorders including IEM, fragmented or absent peristalsis, whereas 57% had normal peristalsis. Another study by Visaggi et al. [60] found that EoE could account for at least 2% of the diagnoses of absent peristalsis in a large cohort of patients who had undergone HRM at a tertiary referral center in the UK. In 2021, a prospective study conducted in a tertiary referral center in Italy found that, of 21 EoE patients undergoing HRM, 52% had abnormal motility, including achalasia, EGJ-OO, hypercontractile esophagus, and DES [61]. Table 1 reports a summary of the studies assessing esophageal motility in EoE.

**Table 1:** Summary of studies assessing esophageal motility in eosinophilic esophagitis

Author and year of the study	Type of study	Type of esophageal manometry	Findings
Landres [35]	Retrospective	CM	1 vigorous achalasia
Attwood [36]	Retrospective	CM	10 NSMD
Hempel [37]	Retrospective	CM	Tertiary contractions at baseline CM and resolution of dysmotility following corticosteroid treatment
Cheung [41]	Retrospective	CM	11 normal peristalsis
Lucendo [38]	Retrospective	CM	1 absent peristalsis
Lucendo [39]	Retrospective	CM	6 NSMD
			3 hypercontractility
			2 normal motility
			1 primary simultaneous waves
Bassett [42]	Prospective	CM	23 normal peristalsis
			5 NSMD
			2 high-amplitude peristaltic waves
Nurko [60]	Retrospective	CM	7 NSMD
			10 normal peristalsis
Moawad [40]	Retrospective	CM	47 normal peristalsis
			25 IEM
			3 NE
Monnerat [43]	Prospective	CM	15 normal peristalsis
			3 IEM
			2 LES dysfunction
Martin [44]	Prospective	HRM (CC V1.0)	6 peristaltic dysfunction
			5 normal peristalsis
			10 PEP
Roman [46]	Retrospective	HRM (CC V1.0)	30 normal peristalsis
			8 weak peristalsis
			5 frequent failed peristalsis
			2 rapid contractions
			1 absent peristalsis
			1 hypertensive peristalsis
			1 functional EGJ obstruction
Savarino [48]	Retrospective	HRM (CC V1.0)	1 achalasia
Clayton [50]	Retrospective	HRM (CC V2.0)	10 normal peristalsis
			1 jackhammer esophagus
			2 weak peristalsis
			1 EGJ-OO
			1 hypertensive LES
Nennstiel [47]	Prospective	HRM (CC V1.0)	12 normal peristalsis
			7 early PEP
			1 CDP
Savarino [52]	Retrospective	HRM (CC V3.0)	20 normal peristalsis
			4 fragmented peristalsis
			3 IEM
			3 EGJ-OO
			2 absent peristalsis
			2 DES
			1 achalasia
Von Arnim [51]	Prospective	HRM (CC V3.0)	11 normal peristalsis
			7 weak peristalsis
			5 EGJ-OO
			1 absent peristalsis
Tanaka [49]	Retrospective	HRM (CC V3.0)	1 jackhammer esophagus
Visaggi [53]	Retrospective	HRM (CC V3.0)	2 absent peristalsis
Ghisa [25]	Retrospective	HRM (CC V3.0)	68 normal peristalsis
			23 IEM
			4 achalasia type 2
			3 achalasia type 3
			5 EGJ-OO
			2 jackhammer esophagus
			1 fragmented peristalsis
			1 absent peristalsis
			1 achalasia type 1
			1 DES
Visaggi [54]	Prospective	HRM (CC V3.0)	10 normal peristalsis
			5 EGJ-OO
			3 hypercontractile esophagus
			2 DES
			1 achalasia

CM, conventional manometry; DES, distal esophageal spasm; EGJ-OO, esophagogastric junction outflow obstruction; HRM, high-resolution manometry; IEM, ineffective esophageal motility; LES, lower esophageal sphincter; NSMD, non-specific motor disorder; PEP, pan-esophageal pressurization; CDP, compartmentalized distal pressurization; NE, nutcracker esophagus

### Clinical implications of esophageal dysmotility in EoE

The chronic uncontrolled inflammation and the structural modifications of the esophagus in EoE alter the function of the organ with progressive dysphagia, food impactions, and, ultimately, stricture formation [62]. In support of this, in a retrospective study on 200 EoE patients [63], it has been shown that a longer diagnostic delay and, consequently, a longer period of active esophageal inflammation, led to an increase in the prevalence of fibrotic features from 46.5% (0–2 years of delay) to 87.5% (> 20 years of diagnostic delay) (*P* = 0.020), with a sixfold increase in strictures prevalence (17.2% at 0–2 years vs. 70.8% at > 20 years; *P* < 0.001). Additionally, the diagnostic delay was found to be the only risk factor for strictures at diagnosis (odds ratio = 1.08; 95% CI 1.040–1.122; *P* < 0.001).

In patients with EoE, obstructive symptoms occur even in the absence of obliterating lesions of the esophagus or when eosinophilic infiltrates are below the threshold for histologic remission [56, 64]. In some instances, the inconsistency between reported symptoms and endoscopy with histology may be the presence of an overlapping motor disorder of the esophagus, which should be investigated on HRM, being dysmotility common in patients with EoE. Accordingly, an HRM should be considered at least in those who do not respond to conventional medical treatment for EoE.

Although esophageal motility of EoE is non-specific [59], characteristic pressure patterns on HRM have been disclosed. PEP and elevated IBP have shown potential to distinguish between EoE patients and controls and segregate the fibrotic from the inflammatory endoscopic phenotype of EoE. Not only these findings help in the characterization of the disease but may also be useful in raising the clinical suspicion of EoE in subjects undergoing an HRM following an unremarkable EGDS, in whom a repeated endoscopy with biopsies could be useful for achieving a final EoE diagnosis.

Dysmotility may be a cause for refractoriness to standard EoE treatment. Although it has been shown that motor abnormalities and symptoms may improve or resolve following medical treatment for EoE [54, 55], it has been reported that those with EoE and achalasia, EGJ-OO, or JE, may require invasive management for symptoms resolution [27, 56]. In this regard, a retrospective study found that pneumatic dilation and/or Heller myotomy achieved symptoms relief in a significant proportion of EoE patients with obstructive motor disorders of the esophagus [27]. Additionally, Tanaka et al. [56] recently reported on an EoE patient with JE experiencing clinical and HRM improvement following per-oral endoscopic myotomy.

In terms of clinical history, dysmotility appears to be a late manifestation of EoE. Consistently, the prevalence of esophageal motility disorders has been found to increase from 36% in those with disease duration of 0–5 years to 83% in those with disease duration ≥ 16 years, confirming that EoE is a chronic and progressive disease [65, 66]. In addition, EoE duration has been identified as a risk factor for esophageal dysmotility (odds ratio of 1.142 per year; 95% confidence interval, 1.004–1.299). Table 2 reports relevant clinical implications of dysmotility in the EoE setting.

**Table 2:** Clinical implications of dysmotility in patients with eosinophilic esophagitis

Clinical setting	Clinical implications
Natural history	Dysmotility may be a tardive manifestation of EoE, as it is esophageal wall remodeling and reduction of esophageal compliance
Diagnosis	Patients may display characteristic pressure patterns on high-resolution manometry which may be helpful to raise the clinical suspicion and investigate EoE in those who do not have preliminary performed an esophagogastroduodenoscopy with esophageal biopsies
Treatment	Dysmotility may be a cause of refractoriness to EoE-specific treatment, and in some cases, invasive management of major motor disorder may be required for symptoms resolution
	Motor abnormalities may resolve following EoE treatment

EoE, eosinophilic esophagitis

## Conclusion

The assessment of esophageal motility in the EoE setting is not taken into consideration in the latest clinical guidelines [6, 7]; however, abnormal esophageal motor function may be relevant in the management of patients with EoE. For this, we reviewed the literature on the pathogenesis, assessment, characteristics, and clinical implication of dysmotility in EoE.

Eosinophils and mast cells can hamper esophageal motility by means of both structural remodeling and myoactive and neurotoxic released mediators, ultimately causing organ dysfunction and predisposing to symptoms and motility disorders.

Although esophageal motility in EoE appears to be variegated and non-specific on both CM and HRM, prolonged esophageal manometry demonstrated a clear association between esophageal symptoms and intermittent dysmotility events [67]. These findings put two arguments under the spotlight: first, symptoms can be related to dysmotility in patients with EoE; second, short-term manometry recordings may miss clinically relevant intermittent dysmotility episodes. This raises questions regarding the suitability of low-volume water-based manometry protocols for the assessment of patients with EoE.

On HRM, EoE-specific esophageal motor patterns have been described, including PEP and elevated values of IBP. Of note, these metrics could segregate EoE patients from controls and distinguish between fibrotic or inflammatory endoscopic phenotype [53, 54, 57]. This has relevant clinical implications as the presence of suggestive HRM features even in the context of a normal EGDS may contribute to raise a clinical suspicion, thus speeding up the diagnostic process and reducing the diagnostic delay in EoE. That is, providing a rationale for a repeated endoscopy with biopsies in those who had undergone a prior unremarkable EGDS during which biopsies had been omitted.

Several studies reported that EoE-specific treatment could lead to improvement or resolution of esophageal dysmotility and symptoms [44, 54, 55], corroborating that eosinophils play a role in the pathogenesis of a clinically relevant functional abnormality. However, other reports showed that invasive management of the motor disorder may be required for symptoms resolution in EoE patients [27, 56], remarking that the management of the disease is complex and may need a combination of both medical, endoscopic, and surgical interventions.

In conclusion, esophageal motor abnormalities may contribute to symptoms, influence treatment refractoriness, and potentially be useful for raising a clinical suspicion of EoE. The assessment of esophageal motility is not currently included in the standard assessment work-up of EoE although recent evidence demonstrated its clinical potential. Further studies should aim at assessing the clinical impact of dysmotility in larger cohorts of EoE patients in a prospective fashion.

## Data Availability

No additional data available.
